# The Calcium Conundrum: Hypercalcemia of Unknown Etiology

**DOI:** 10.7759/cureus.91200

**Published:** 2025-08-28

**Authors:** Alp S Kahveci, Lindsay Moy, Mohamed M Eletrebi, Brittany Bettendorf

**Affiliations:** 1 Internal Medicine, University of Iowa Hospitals and Clinics, Iowa City, USA; 2 Internal Medicine/Rheumatology, University of Iowa Hospitals and Clinics, Iowa City, USA; 3 Pathology, University of Iowa Hospitals and Clinics, Iowa City, USA

**Keywords:** 1.25- dihydroxy vitamin d, bilateral lower limb weakness, bone marrow biopsy, bone marrow granuloma, granulomatous disease, hypercalcemia, parathyroid hormone (pth), sarcoidosis, systemic glucocorticoids

## Abstract

Hypercalcemia is a common clinical finding with a wide variety of clinical presentations and etiologies. The differential is broad and includes hyperparathyroidism, malignancy, medication adverse effects, endocrinopathies, and a variety of granulomatous diseases. We present the case of an 84-year-old woman with severe parathyroid hormone-independent hypercalcemia and elevated 1,25-OH Vitamin D. Non-necrotizing granulomas were identified on bone marrow biopsy. After an exhaustive workup for granulomatous disease, the patient was felt to have sarcoidosis isolated to the bone marrow and started on prednisone to suppress 1-alpha hydroxylase activity.

## Introduction

Hypercalcemia is a common finding in the emergency department, ranging between 0.7% to 7.5% of visits [1-4]. Manifestations are diverse, including gastrointestinal symptoms, muscle weakness, neuropsychiatric changes, bone pain, and fatigue.

Workup begins with evaluating for primary hyperparathyroidism, the most common cause of hypercalcemia. Nearly 80% of cases of primary hyperparathyroidism are secondary to adenoma formation in a single parathyroid gland [5]. Risk factors include head and neck radiation, as well as long-standing lithium use.

Patients with a parathyroid hormone-independent process warrant further assessment. Paraneoplastic processes are the second most common etiology of hypercalcemia [5], often developing from the secretion of parathyroid hormone-related protein (PTHrP) in squamous cell carcinoma or adenocarcinoma [5,6]. PTHrP promotes the synthesis of receptor activator of nuclear factor-kappa B ligand (RANKL), thereby increasing bone resorption [7]. Metastatic disease, including breast cancer, multiple myeloma, and Hodgkin and non-Hodgkin lymphoma, may precipitate hypercalcemia through a cascade of inflammatory cytokines that stimulate RANKL [7].

Other etiologies of parathyroid hormone-independent hypercalcemia include exogenous vitamin D intoxication, medication adverse effects, endocrinopathies, immobility, and, less commonly, granulomatous diseases [5].

Granulomatous disease is an infrequent finding on bone marrow biopsy, with an incidence estimated between 0.3% and 2.2% [8]. Hypercalcemia is driven by increased macrophage production of 1-alpha-hydroxylase, converting inactive vitamin D into the active form [5]. Given the broad differential for granulomatous disease, a precise diagnosis may be challenging. Here, we present a case of recurrent, symptomatic hypercalcemia from sarcoidosis isolated to the bone marrow with clinical response to glucocorticoid therapy.

## Case presentation

An 84-year-old female, with a history of paroxysmal atrial fibrillation, heart failure with preserved ejection fraction, and chronic kidney disease stage IIIa, presented to the emergency department for lower extremity weakness and cramping pains resulting in recurrent falls. On further review, she reported three recent hospital visits for similar concerns with labs concerning for moderate hypercalcemia (maximum Ca2+ of 13.3 mg/dL; reference range, 8.5-10.5 mg/dL) with an appropriately suppressed parathyroid hormone (PTH) level. Workup during those visits was negative for multiple myeloma. She denied any recent history of surgery or period of immobility.

On admission, labs were remarkable for Ca2+ 15.3 mg/dL (reference range 8.5-10.5 mg/dL, Table 1), with ionized Ca2+ 7.1 mg/dL (reference range 4.57-5.43 mg/dL). PTH was appropriately low at 6 pg/mL (reference range 15-65 pg/mL), and PTH-related peptide was 0.8 pmol/L (reference range less than or equal to 4.2 pmol/L). 25-OH vitamin D was in the normal range at 51 ng/mL (reference range 20-80 ng/mL), while 1,25-OH vitamin D was elevated at 152 pg/mL (reference range 19.9-79.3 pg/mL). An angiotensin-converting enzyme level returned elevated at 114 u/L (reference range, 16-85 u/L). Her creatinine was elevated to 3.22 mg/dL, up from a baseline of 1.2 mg/dL. Over the preceding three months, she had developed a chronic pancytopenia with white blood cell count as low as 1.7 K/mm3 (reference range 3.7-10.5 K/mm3), hemoglobin nadir of 9.6 g/dL (reference range 11.9-15.5 g/dL), and platelet nadir of 117 K/mm3 (reference range 150-400 K/mm3).

**Table 1:** Comprehensive laboratory workup

Lab Test	Result	Reference Range
Calcium (Ca²⁺)	15.3 mg/dL	8.5 – 10.5 mg/dL
Ionized Calcium (Ca²⁺)	7.1 mg/dL	4.57 – 5.43 mg/dL
Parathyroid Hormone (PTH)	6 pg/mL	15 – 65 pg/mL
PTH-Related Peptide (PTHrP)	0.8 pmol/L	≤ 4.2 pmol/L
25-OH Vitamin D	51 ng/mL	20 – 80 ng/mL
1,25-OH Vitamin D	152 pg/mL	19.9 – 79.3 pg/mL
ACE (Angiotensin-Converting Enzyme)	114 U/L	16 – 85 U/L
Creatinine	3.22 mg/dL	0.51 – 0.95 mg/dL
WBC Count	1.7 K/MM³	3.7 – 10.5 K/mm³
Hemoglobin	9.6 g/dL	11.9 – 15.5 g/dL
Platelet Count	117 K/MM³	150 – 400 K/mm³

**Figure 1:**
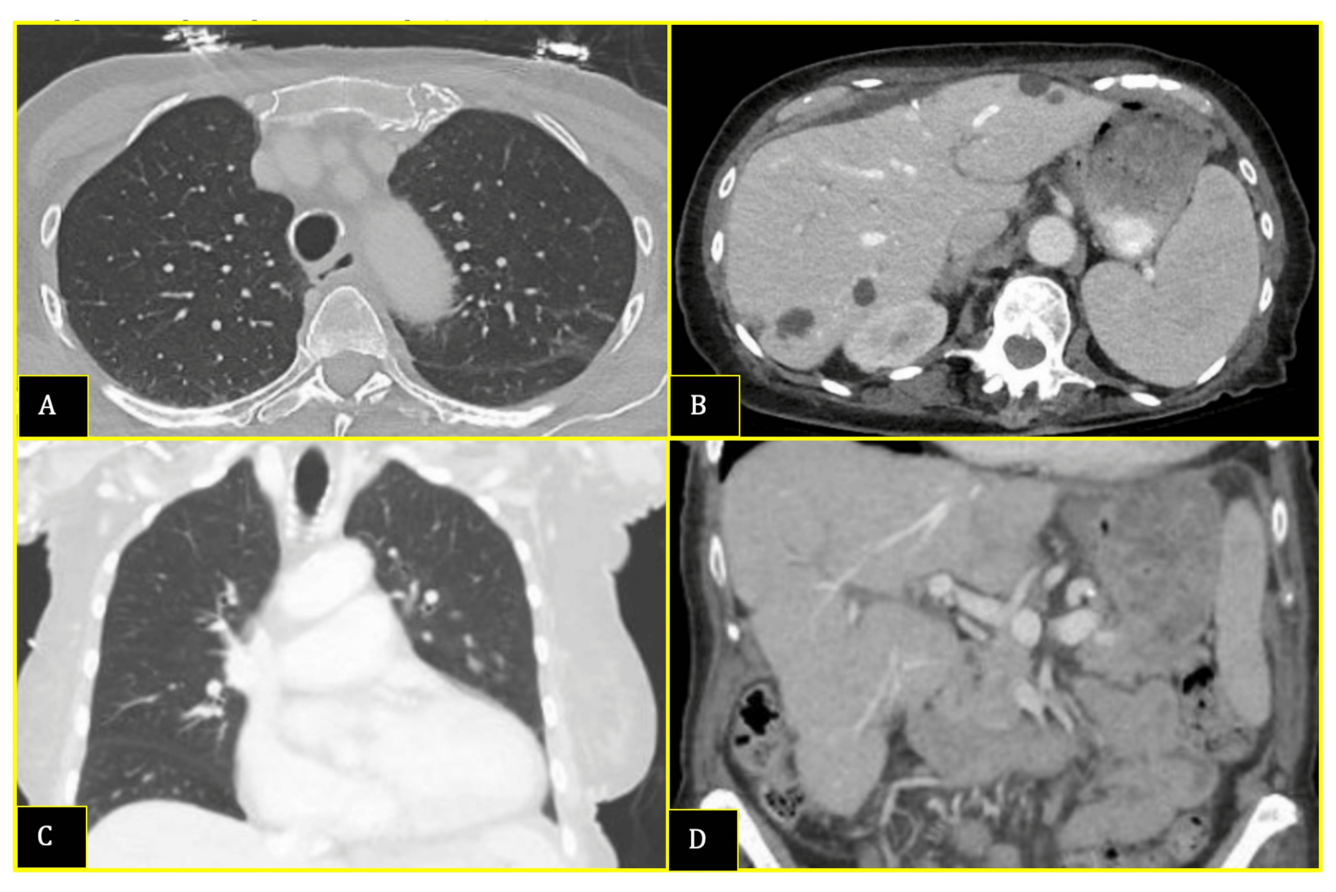
Axillary and coronal cross-sectional planes of the chest, abdomen, and pelvis A CT of the chest (A and C), abdomen, and pelvis (B and D) with contrast demonstrated cardiomegaly, coronary artery and thoracic aortic atherosclerotic calcifications, hepatomegaly (18.7 cm craniocaudally) with scattered cysts, and splenomegaly (15.4 cm craniocaudally). There was no appreciable lymphadenopathy or masses.

**Figure 2:**
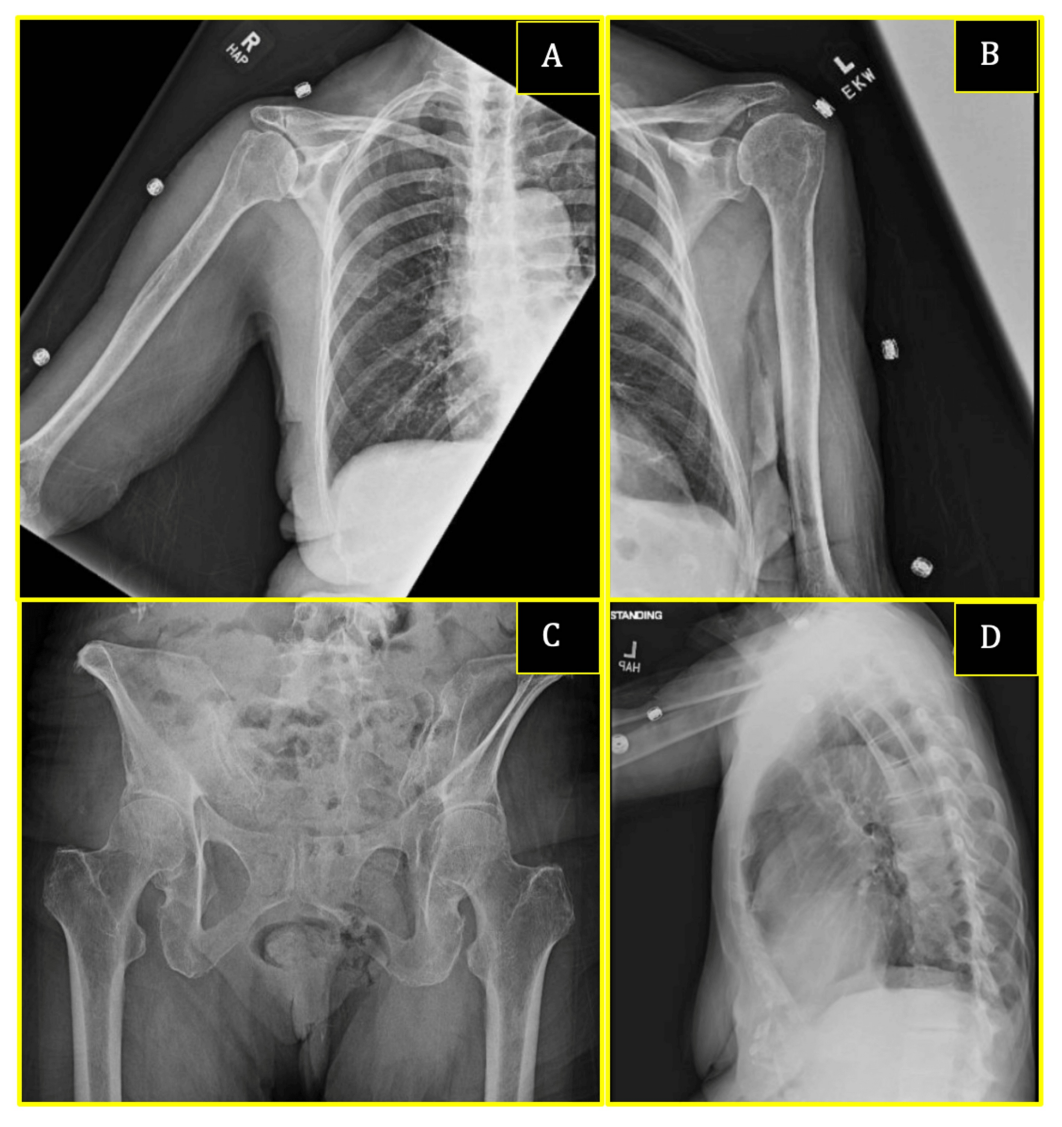
Complete skeletal survey No focal lytic lesions concerning for malignancy were identified throughout, including the right upper extremity (A), left upper extremity (B), pelvis (C), and rib cavity (D). Diffuse osteopenia is noted, with scattered degenerative disease throughout the spine.

Given PTH-independent hypercalcemia with inappropriately elevated 1,25-OH vitamin D, a malignancy workup was pursued. Computed tomography of the chest/abdomen/pelvis was negative for lymphadenopathy or masses (Figure 1), while a skeletal survey was negative for lytic lesions (Figure 2). Repeat serum and urine protein electrophoresis did not demonstrate monoclonal proteins.

**Figure 3:**
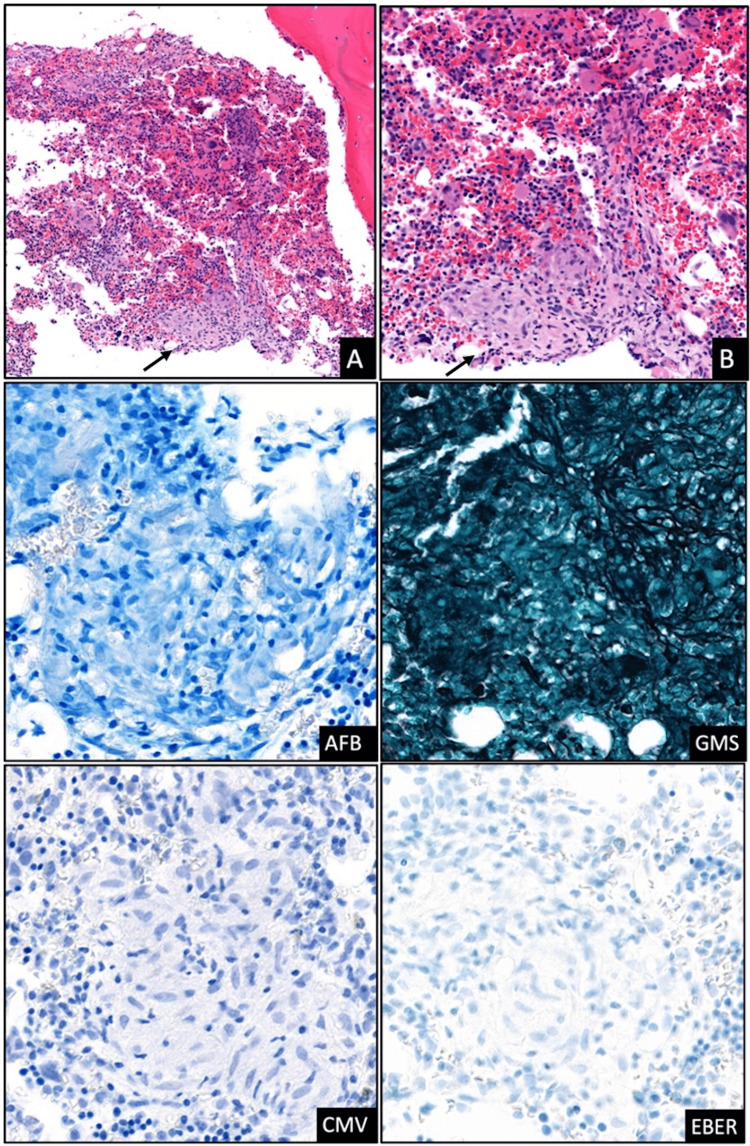
Bone marrow biopsy Hematoxylin and eosin (H&E) staining of the bone marrow core biopsy at a low (10X; A) and high (20X; B) power magnification, illustrating non-necrotizing granulomas (arrows) admixed with hematopoietic elements. Acid-fast bacilli (AFB) and Grocott-Gomori's methenamine silver (GMS) staining were negative for mycobacterial or fungal organisms. Cytomegalovirus (CMV) immunohistochemical stain was negative, as was the Epstein-Barr virus (EBV) EBER (Epstein-Barr virus–encoded RNA) study.

In the setting of chronic pancytopenia, a bone marrow biopsy was obtained to rule out a hypo-proliferative process. The bone marrow biopsy report noted multiple foci of non-necrotizing granulomas with negative fungal and mycobacterial stains, negative cytomegalovirus (CMV) staining, and negative Epstein-Barr virus (EBV) EBER (Epstein-Barr virus-encoded RNA) study, and no evidence of plasma cell neoplasm, myelodysplastic syndrome, nor histiocytic neoplasm (Figure 3).

Rheumatology and infectious disease services were consulted. The patient’s physical exam was unrevealing. A broad infectious workup was completed, returning negative for histoplasmosis, blastomycosis, coccidiomycosis, (1,3)-beta-D-glucan, human immunodeficiency virus, hepatitis B and hepatitis C virus, CMV, EBV, syphilis, Brucella spp., Bartonella spp., Coxiella burnetti, and Mycobacterium tuberculosis.

**Figure 4:**
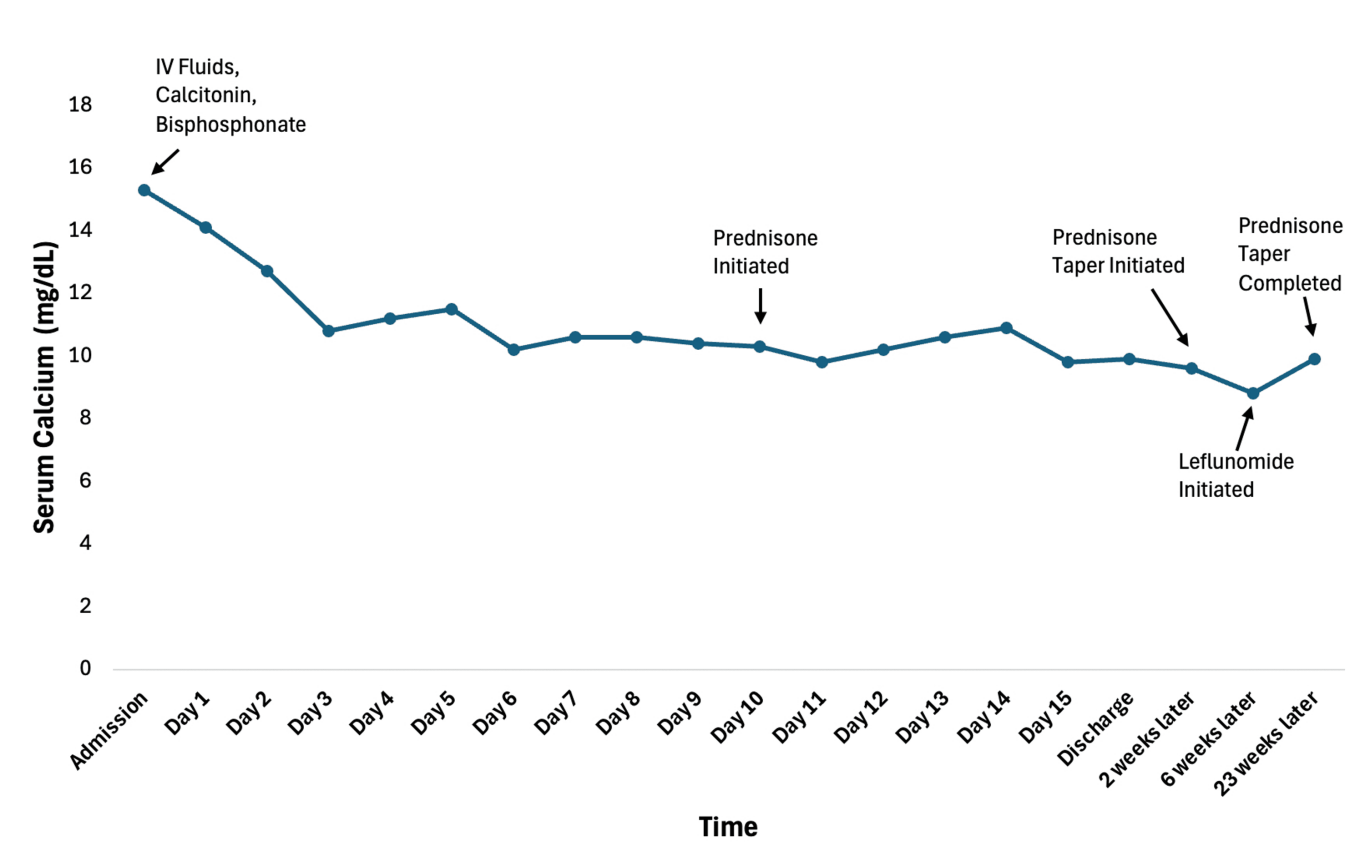
Trend in serum calcium Hypercalcemia swiftly corrected in the inpatient setting in response to therapeutic interventions. At the time of discharge, the patient was normocalcemic while on prednisone therapy. Several months after initial presentation, she remained normocalcemic on a slow prednisone taper.

With an exhaustive workup demonstrating no definitive etiology of the bone marrow granulomas, our patient was started on high-dose prednisone for treatment of an atypical presentation of sarcoidosis isolated to the bone marrow. She was discharged home after an interval improvement in serum calcium and symptoms. On follow-up several weeks later in the rheumatology clinic, she had sustained resolution of hypercalcemia and was started on leflunomide as a steroid-sparing agent (Figure 4). Importantly, the patient tolerated the slow taper of prednisone without any recurrence of symptoms, with monitoring labs remaining stable on follow-up several months later.

## Discussion

The differential diagnosis for granulomatous bone marrow disease is quite broad, including malignancy, infection, drug-associated, connective tissue disease, and sarcoidosis.

Malignant neoplasms are commonly identified as the precipitating event of bone marrow granulomas [8]. While Hodgkin and non-Hodgkin lymphomas are frequently noted, non-hematologic malignancies, such as ovarian, urothelial, and colonic carcinomas, are also described [8,9]. Infectious etiologies include tuberculous and nontuberculous mycobacteria, brucellosis, disseminated fungal infections, EBV, and CMV. Drug-associated granulomas are far less common, with amiodarone, procainamide, and sulfonamides serving as culprits [8,9]. Ultimately, as many as 13% of cases of bone marrow granulomas remain without a known cause [8]. The long-term prognosis is often favorable in those patients [9].

Infiltration of the bone marrow is a rare feature of sarcoidosis [10,11]. Classic illness scripts depict a younger female patient with respiratory symptoms and imaging findings of bilateral hilar adenopathy [12]. Nearly half the patients have extra-pulmonary manifestations, the earliest of which include vision changes, rash, and inflammatory arthritis. However, these findings may also be absent, as seen here.

Hypercalcemia in granulomatous diseases like sarcoidosis is mediated through increased macrophage activity of 1-alpha-hydroxylase. Notably, glucocorticoids suppress the activity of 1-alpha-hydroxylase, rapidly correcting hypercalcemia [13]. Glucocorticoids remain the first-line agent for treatment of pulmonary sarcoidosis and extrapulmonary disease, including bone sarcoidosis, given the evidence for prevention of disease progression and improvement of quality of life [14-16]. Cases of steroid-refractory hypercalcemia have been reported, with patients often responding to ketoconazole (a potent inhibitor of 1-alpha-hydroxylase and other cytochrome P450 enzymes) [17,18].

Given concerns for significant adverse effects from prolonged steroid therapy (such as osteoporosis in an elderly female), consideration is often given for the initiation of steroid-sparing agents. Antimetabolites like methotrexate, azathioprine, or leflunomide are second-line therapies, with anti-tumor necrosis factor agents like infliximab serving as third-line [14,18,19]. Here, methotrexate was not favored due to its toxicity profile and the patient’s renal injury, while the risk for azathioprine-related arthralgias and vision changes contributing to falls was considered. In a small study, leflunomide was found to be effective in the treatment of patients with sarcoidosis intolerant to methotrexate, and in another study, 83% of patients with extrapulmonary disease had a partial or good response to therapy [20,21]. After carefully weighing the adverse effects against the above context, leflunomide was ultimately selected as our patient’s steroid-sparing agent.

## Conclusions

In cases of hypercalcemia where initial workup is unrevealing for primary hyperparathyroidism or malignancy, clinicians should maintain a high index of suspicion for granulomatous diseases. Here, we highlight an exceedingly rare case of isolated bone marrow granulomas and the importance of keeping a broad differential. Ruling out other etiologies of granulomatous bone marrow diseases was imperative to narrowing our patient’s differential diagnosis to sarcoidosis and initiating timely therapy. Glucocorticoids remain the first-line therapy for sarcoidosis, effectively lowering calcium levels by suppressing inflammation and vitamin D synthesis.
